# The Health Ministry's Progress

**Published:** 1922-07

**Authors:** C. Addison

**Affiliations:** (First Minister of Health)


### THE HEALTH MINISTRY'S PROGRESS.

By the RT. HON. C. ADDISON, M.D., M.P. (First Minister of Health).

THREE years ago, on July 1, the whole of the powers and duties of the Local Government Board, the Insurance Commissioners of England and Wales, various health duties hitherto discharged by the Home Office and the Board of Education as specified in the Act, together with certain powers of the Privy Council, were transferred to the Ministry of Health. But in years past far-sighted men like the late Sir Robert Morant had been trying to concentrate the main health services of the country in a single department, instead of some 20 offices overlapping each other in the discharge of their medical duties.

Early in 1917, at the request of the late Lord Rhondda, I acted as chairman of a committee which reported on the project and submitted definite proposals. From that time onwards, as Minister of Reconstruction, I pressed the Government to take measures to husband and strengthen the health of the people. The haphazard and conflicting organisations in Whitehall meant waste of money, confusion of effort, and continued physical inefficiency. There was need of a concerted offensive on what John Bunyan calls "the captains of the men of death." For two years these efforts to economise in administration and to extend for all the frontiers of life were opposed both within and outside the Cabinet. But the undoubted facts of our impaired physique were so overwhelming that, after a two years' fight behind the scenes, the Ministry of Health was founded. It was a reform in the machinery of government, an attempt to bring a clear and single purpose into our scattered and often conflicting efforts, and can "be judged only in its working and over a long period of time." The growth of medical science has been slow, and we built at the Ministry for the future. In 1950 it will be possible to judge whether the officials, who tried to apply the lessons of preventive medicine to the health of a nation, have built wisely and well.

It is easy in dealing with so vast a subject to become clogged with detail. The functions of a Ministry of Health range from measles to milk, from rates to reservoirs, from cottages to canal boats. I can only indicate the general progress made, and must resist the temptation to stray into the mazes of national insurance or the jungle of local government.

There has recently been some questioning as to the necessity of such a Ministry. Apart from the need to avoid the waste and overlapping that resulted from a score of departments trying to carry out the functions that rightly belong to one central Ministry, the state of the national physique and health demanded attention. The Health Ministry was not the child of the optimism of the first year of peace. It was conceived by men, some of a past generation, who recognised that the health and physique of the people is the principal asset of a nation. It was born after prolonged labour, much thought, a half-century of experiments in public health, and a volume of evidence that our national health was poorer than it should be. Who can gainsay the evidence? Ten years' medical inspection in the public elementary schools proved that over a million children of school age were so physically or mentally impaired as to be unable to receive reasonable benefit from the education which the State provided. National Insurance statistics proved that each year insured persons lost a minimum of 14,000,000 weeks of time, a period roughly equal to 270,000 years. The medical examination of recruits, the exact figures for which need qualification, told a similar story of ill-health.

### Infant Welfare.

The health of the mother and child must obviously be the basis of national health, and I rejoice at the fall of the infant mortality rate from 156 per 1,000 in 1896-1900 to 80 per 1,000 in my last year of office at the Ministry. Sir Alfred Mond, after an unfortunate wobble on the question of milk for infants, now consistently supports the policy of maternity and child welfare that we initiated in 1919. The Geddes Committee, which in practically every respect gave its approval to the public health services for which I was responsible, has recommended that there shall be no diminution in the nation's work for the babi s. But constant vigilance is the price of safety of the infants. Unemployment is already lowering their vitality. A hot summer may mean death to thousands. Mothers are glad enough to carry out the advice of those who know, if they can and if they understand it so far as they can with homes still inadequate, and I regard the vastly improved system of instruction given by the local authority agencies, and by the maternity and child welfare agencies, as one of the most important causes of the recent improvement. But the increase of the numbers of deaths of mothers due to childbirth is disquieting.

One immediate result of the formation of the Ministry of Health is that on its third birthday, for the first time, the physical care of mother, baby and child is supervised by one Department. There is at last continuous responsibility from birth upwards for each child born.

### World Plagues.

Out of the mass of diseases against which the new Ministry directs the weapons of preventive medicine, I can only select certain examples in order to show the extent and value of its work. Since the Armistice there have been plagues of cholera, typhus and yellow fever throughout the world, and especially in Eastern Europe. Owing to the efficiency of our Port Sanitary Authorities, these scourges of mankind have not gained a foothold in our islands. The risk of invasion by these diseases carried on ships in 1919 caused me grave anxiety. We obtained grants to help Port Sanitary authorities, and arranged centrally for the furnishing of special expert advice and assistance whenever required. On many occasions this machinery was used with remarkable promptitude, Dr. Reece and his medical officers were such reliable watchmen at the gates that only a few cases slipped through, and these were quickly traced and isolated. If it had not been for the watchfulness of these experienced men, we might well have experienced an epidemic of cholera, unknown in this country since 1866, and of typhus or gaol fever.

Much more complete arrangements were also made to control malaria, yellow fever, and the other tropical diseases. In the building in Parliament Street sit medical men who enable us as a nation to fulfil our Imperial health duty in India, in Africa and in our Dominions. When I went to the Local Government Board early in 1919, these responsibilities were dimly appreciated. The arrangements to collect information on which action could be taken were incomplete and defective. Sir George Buchanan, whose immense services to the world's health were honoured in the New Year, has established a central clearing house of information in Whitehall. He has formed admirable arrangements with the Foreign Office, the Colonial Office and the India Office, so that early and accurate intelligence of infectious diseases in all parts of the world is received at the Ministry of Health. Now he is extending his work to the international health organisation developing in connection with the League of Nations, and it is gratifying to know that the scheme of the Health Section of the League was largely drafted in our own Ministry.

But early information of disease and central organisations are incomplete unless there is opportunity for investigation and the acquisition of new knowledge. Medical research has, therefore, been made freely available for all home departments and for all Dominions and Crown Colonies, but is under the Privy Council and free from all Departmental interests. The result has already abundantly justified this scheme of work. The Government has allocated £150,000 instead of £50,000 to the Medical Research Council. This body, which is magnificently served by its Secretary, Sir Walter Fletcher, works in close co-operation with the Health Ministry, which restricts its own investigations to problems of immediate practical interest arising out of every-day administration.

### Lunacy Reform.

Three years ago the Ministry became legally interested in the enactments relating to lunacy and deficiency, but the actual transfer of powers did not take effect till May 17, 1920. No change was made in the constitution or procedure of the Board of Control, but the Minister of Health became responsible to Parliament, and the treatment of mental diseases was more closely co-ordinated with that of other diseases.

Much of the present mental disease is directly preventible. Conditions of life and industry are to-day creating lunatics at an average rate of 22,000 certified cases a year, who cost over £9,000,000 annually. There are in addition the 150,000 cases of the feeble-minded. The *early* treatment of mental disorders is vital to the question, as was well shown at a conference this year presided over by Sir Frederick Willis, provision for such treatment was made in the Additional Powers Bill, which was accepted by the Commons and rejected by the Lords. Ignorant opposition of the anti-waste Press in this case led to much unnecessary expenditure of public money, but I am glad to see that Sir Alfred Mond is wisely continuing the policy laid down in that ill-fated measure. Mental disorder is both a medical and a social problem. It can be checked more by preventing its occurrence and by treatment in the early stages than by improved methods of treating confirmed lunacy. Few results were more definite and gratifying in the medical history of the war than those obtained from the early and scientific treatment of mental disorders. If we were allowed to apply them they would free thousands from the life-long handicap of certified insanity and save vast sums in costly buildings and maintenance. We must possess our souls in patience, well knowing that the " anti-wasters " will be found out in due time.

I have dealt only with a small part of the duties of the new Department. Volumes will in time be filled with scientific descriptions of this new piece of British constructive work that is being copied by France, Czecho-Slovakia, Poland, Australia, Canada, South Africa, Brazil, Siam, and the Republic of Cuba. The facts, however, prove the progress already made. After two years of a Health Ministry, the infant mortality rate in 1920 of 80 per 1,000, and of 75 per 1,000 in London, was the lowest on record. The general death-rate (12·4) showed a decline at most ages, and gave an increased expectation of a longer life to everyone. The birth-rate in 1920 rose to 25·4. Tuberculosis continued to fall in notifications and deaths certified. Although the various factors that brought about these records cannot be differentiated, they represent triumphs of preventive medicine. Despite the progress made in almost every aspect of national health, let it never be forgotten both by the Ministry's friends and enemies that so far it has only made its childish first steps. Mistakes have been inevitable. Foolish expectations that in a few months the health of everyone would be improved have been disappointed. There have, of course, been no dramatic results achieved in a short time. The pictures highly coloured for electioneering purposes in 1918 will not be ready for the public view for years to come.

Much of real value, not possibly of importance, to the " stunt " politician, but in fact a steady evolution, has been carried out. There are men of constructive power—elder statesmen in outlook, young men in enthusiasm—at the Ministry of Health and on its councils who are steadily working so that human life in the future may be potentially better than in the past.

